# Smoking or My Job? US Media Coverage of Nonsmoker-Only Hiring Policies

**DOI:** 10.1371/journal.pone.0144281

**Published:** 2015-12-03

**Authors:** Patricia A. McDaniel, Brie Cadman, Naphtali Offen, Ruth E. Malone

**Affiliations:** Department of Social and Behavioral Sciences, School of Nursing, University of California San Francisco, San Francisco, California, United States of America; University of Tennessee Health Science Center, UNITED STATES

## Abstract

**Objectives:**

Media advocacy plays a critical role in tobacco control, shaping the content of news in ways that generate public support for tobacco control. We examined US media coverage of nonsmoker-only hiring policies, which have little US public support, exploring the extent to which tobacco control advocates and experts have engaged the media on this controversial issue.

**Methods:**

We searched online media databases (Lexis Nexis, Access World News, and Proquest) for articles published from 1995–2013, coding retrieved items through a collaborative, iterative process. We analyzed the volume, type, provenance, prominence, content and slant of coverage.

**Results:**

We found 1,159 media items on nonsmoker-only hiring policies, most published in local newspapers in regions where such policies were enacted. The most common reason given for implementing such policies was to reduce healthcare costs. Most news items offered reasons both to support and oppose such policies; thus, the slant of the majority of news items was neutral or mixed. Tobacco control advocates or experts were infrequently cited or quoted in news items, and rarely authored opinion pieces. Those who expressed opinions were more likely to support than oppose nonsmoker-only hiring policies, for economic and health reasons. Ethical concerns about the policies were seldom raised.

**Conclusions:**

As presented in the media, nonsmoker-only hiring policies were primarily framed in terms of business cost savings and had little connection to health initiatives. Tobacco control advocates were rarely quoted and their positions were not consistent. Given their intrusiveness and the lack of strong evidence that such business policies actually do improve worker health, tobacco control advocates may feel that the status quo is preferable to engaging on a policy that the majority of Americans dislike.

## Introduction

Tobacco control is highly newsworthy in the US [1], and the media play key roles in advancing the tobacco control agenda [2, 3]. By choosing what issues to cover and how to cover them, news coverage can shape the public’s perception of tobacco issues [2, 4–6, 7], p. 52, [8–10]. News media also offer forums for members of the public and “experts” to express their views, make claims, convey information, and offer solutions about tobacco issues [1, 2]. Tobacco control advocates have used a variety of media advocacy techniques to promote media coverage that will generate public support for tobacco control [11–13].

While many workplace-related tobacco control policies, such as smokefree environments [14], have strong public support in the US, others are more controversial. Creating “smoker free workplaces” is one such policy. Formal nonsmoker-only hiring policies first appeared in the US in the 1980s. Some were aimed at firefighters and police officers, and were intended to reduce future disability payments by municipal governments, by weeding out, for example, firefighters whose lung disease was smoking-related rather than fire-related [15, 16]. In 1985, a hospital in Illinois became one of the first healthcare institutions to hire only nonsmokers, citing a desire to demonstrate “leader[ship] in cutting down smoking” [17]. In response, the tobacco industry and its paid ally, the American Civil Liberties Union (ACLU), aggressively promoted so-called “smokers’ rights”/smoker protection laws in state legislatures [18, 19]. Twenty-nine states and the District of Columbia have passed such laws, the majority from 1990–1994 [20]. However, in the remaining 21 states, employers may choose not to hire people based on their smoking status. In 2013, in a survey of large employers, 4% reported not hiring smokers, with hospitals the most likely type of employer to do so [21]. Eighty-six percent of Americans disapprove of such decisions, a proportion that has not changed over the last decade [22].

Public health advocates’ and experts’ views of nonsmoker-only hiring policies are divided. Some regard them as appropriate tools to model healthy practice or to encourage smokers to quit, a form of benevolent paternalism [23, 24]. Others argue that they are punitive, ineffective at promoting smoking cessation, and unethical because, with smoking concentrated among lower socioeconomic status groups, they disproportionately affect an already disadvantaged population [25–27]; moreover, some argue that nonsmoker-only hiring policies are inconsistent with healthcare institutions’ ethical norms, which include caring for those whose poor health may be partly self-inflicted [26]. The extent to which public health advocates on either side have successfully engaged the media on this issue is unknown, however, as no previous studies have examined media coverage about the topic. In this paper, we explore whether nonsmoker-only hiring has been portrayed as a public health or tobacco control issue, and whose perspectives were reflected in media accounts.

## Methods

We searched three online media databases (Lexis Nexis, Access World News, and Proquest) for US news items published between 1995 (the year after the majority of states had passed smoker protection laws) and 2013 that focused on employers who had chosen to no longer hire smokers. The three databases covered 1,381 news sources, including 999 local and national newspapers, 11 magazines, 61 newswires, 256 web-only news sources, 53 television network news broadcasts, and National Public Radio. We used a variety of search terms to locate news items, starting with general terms intended to capture all employers who had implemented this policy (e.g., smokers AND (employ OR hire OR job)). We used retrieved items to identify more specific search terms (e.g., the names of particular employers who had stopped hiring smokers). We stopped searching once no new items were found. In order to understand the reach of media coverage, we included items with nearly-identical content that were published in multiple news outlets.

We coded news items through a collaborative, iterative method. We created an initial coding sheet after reviewing 27 news items; it was an adaptation of a coding sheet drawn from an earlier project examining news coverage of retailers who had voluntarily ended tobacco sales [28]. After discussion, we refined and edited it and drafted coding instructions. Next, two coders (the second and third authors) independently coded an overlapping set of 23% (n = 265) of the items (chosen with a random number generator), checking in with one another and the first author early in the process to compare results, discuss discrepancies, and refine coding instructions.

We assessed inter-coder reliability of the overlapping sample using Gwet’s AC1 statistic, an improvement on the kappa (κ) statistic, which becomes unreliable without adequate coding variety [29]. For example, if the correct code is “no” 90% of the time for one item, the resulting κ value is low even when inter-rater agreement is high [30–32]. Like the κ statistic, the value of AC1 ranges from 0–1, and can be interpreted in a similar manner. For the overlapping sample of 265 items, all of the non-static variables achieved Gwet’s AC1 values of .66 or greater. Average inter-coder reliability for all non-static variables was 0.88.

After confirming inter-coder reliability with the overlapping sample, each coder independently coded one-half of the remaining (randomly assigned) news items. We also recoded the items coded early on to be consistent with codebook revisions. We coded story characteristics (i.e., news source, story type, date, accompanying photo, page number, word length, etc.) and content. Allowing for multiple mentions, we coded for the presence (“yes”) or absence (“no”) of content; for the purposes of this paper, we focused our analysis on content related to arguments for and against the nonsmoker-only hiring policy and the sources and evidence relied upon. We defined public health or tobacco control advocates as representatives of organizations such as the American Cancer Society that promote or support tobacco control policies, and tobacco control experts as academics working in the field of public health or tobacco control. Because the items collected were not a random sample and we are not extrapolating from them, no significance testing was done [33]. Rather, we report the findings from the entire population of items meeting the search criteria.

This study has limitations. Although they covered a large number of national and local newspapers, the news databases we searched are not comprehensive. Moreover, our search terms may not have been exhaustive; thus, we may not have identified and included all relevant news items in our study. We chose to include nearly identical content published by different news sources in order to capture the breadth of news coverage; excluding near duplicates would not have allowed us to assess how far these news items “traveled” throughout the US, and how many times the issue was covered. As a result, any similar content was coded multiple times. Therefore, our findings reflect *all* coverage that appeared, not *unique* stories.

## Results

We found 1,159 US news items published from 1995 to 2013 concerning employers adopting a non-smoker only hiring policy. The majority of employers (840, 72.5%) applied the policy only to new hires, exempting current employees. Healthcare providers and “health-focused” organizations (e.g., the World Health Organization and local departments of public health) were the most frequently mentioned type of employer with this policy (386, 33.3%); other types mentioned included governments (211, 18.2%), health benefit providers (126, 10.9%) and fire and police departments (59, 5.1%) (Table 1).

**Table 1:** News Items (n = 1,159) on employers with policies to hire only nonsmokers: United States, 1995–2013.

Variable	No.	(%)
News source		
Local newspaper	914	78.9
National newspaper^a^	36	3.1
News wire/service	95	8.4
Web based	61	5.3
TV news	34	2.9
Radio	13	1.1
Magazine	6	0.5
Story type		
News	753	65.0
Editorial or op-ed	240	20.7
Letter to the editor	166	14.3
Section (newspapers only, n = 923)		
Front page	106	9.1
First page of section	107	9.2
Photo	170	14.7
Geographic region		
West	117	10.1
Midwest	192	16.6
South	403	34.8
Northeast	203	17.5
National^b^	244	21.1
Employer type		
Healthcare provider/health focused organizations	386	33.3
Government	211	18.2
Health benefit providers	126	10.9
Uniformed personnel (police/fire)	59	5.0
Multiple types	162	14.0
Not specified	114	9.8
Other^c^	101	8.7

^a^The *New York Times*, *Wall Street Journal*, *Washington Post*, *Los Angeles Times*, *Christian Science Monitor*, and *USA Today*

^b^News items published in national newspapers, magazines, or on the web, and news items broadcast by National Public Radio or by national television news (CNN, NBC, CBS, FOX, and ABC).

^c^Includes employers such as Alaska Airlines, Hollywood Casino, Union Pacific, and Scotts Miracle-Gro.

Most news items (914, 78.9%) were local newspaper articles (daily or weekly newspapers serving a specific city or region, such as the *San Francisco Chronicle*), but items also appeared in national newspapers (newspapers, such as the *New York Times*, that circulate throughout the US), news wires and services, web sites, magazines, and TV and radio programs (Table 1). News stories constituted the majority of items (753, 65.0%)(Table 1). Tobacco control advocates and experts wrote a handful of opinion pieces or letters to the editor (9 of 406); nearly half (4) of the authors were representatives of local branches of the American Cancer Society. Item length ranged from 7 to 7,572 words, with a median of 479 words.

**Fig 1:**
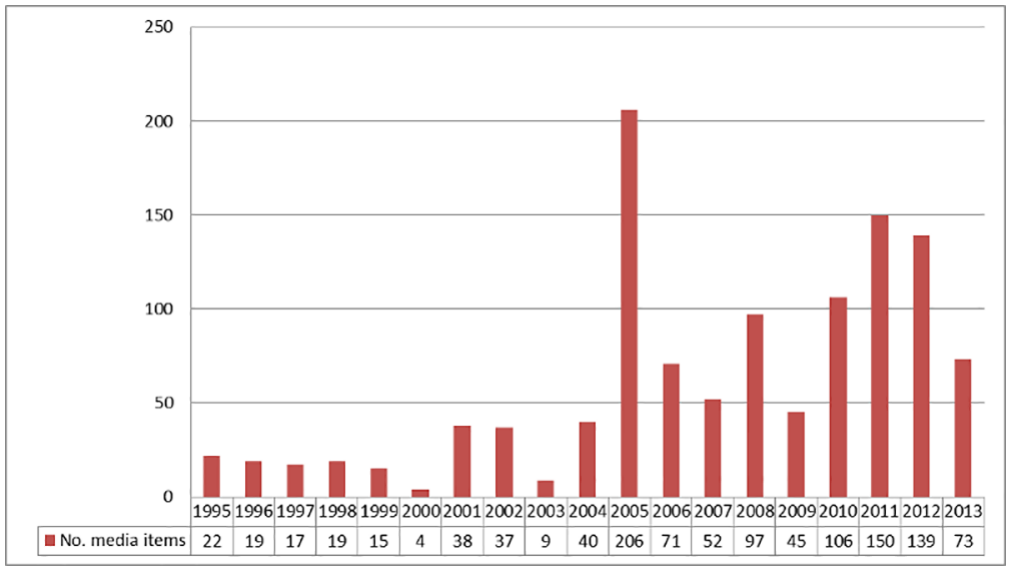
Number of news items about nonsmoker-only hiring policies, by year: United States, 1995–2013 (n = 1,159)

The volume of news coverage varied between 1995 and 2013 (Fig 1). Four years accounted for the majority (601, 51.9%) of coverage: 2005, 2010, 2011, and 2012. In each of those years except 2010, the surge in coverage could be attributed to particular employers generating extensive media attention. For example, in 2005, more than half of news items (113 of 206) focused on Weyco (now Meritan Health), a Michigan-based medical benefits provider. That year, Weyco updated its previously established nonsmoker-only hiring policy (one that had generated no media coverage) to require *all* employees to be tested for nicotine; if employees did not take the test or tested positive, they would be dismissed. Four employees refused to take the test and were fired [34]. In 2011 and 2012, Pennsylvania-based Geisinger Health Systems received the most attention of all named employers (72 of 289 items). In 2011, it announced that the following year it would no longer hire smokers, with current employees exempt from the policy. Despite not being the first (nationally or locally) [35, 36] or the largest [37, 38] healthcare provider to adopt such a policy, it received extensive media coverage.

Coverage of employers adopting nonsmoker-only hiring policies varied by region (Table 1). Over one-third of news items (403, 34.8%) were published in the South (Table 1). This was largely due to widespread coverage in Florida (228), where numerous local government offices (e.g., Sarasota County, and the cities of St. Cloud, Temple Terrace, and North Miami) proposed or passed nonsmoker-only hiring policies. News outlets in the Northeast accounted for nearly 20% (203, 17.5%) of all news items (Table 1), with Pennsylvania, the home state of Geisinger, producing more than half (129, 63.5%) of these items.

In newspapers, issues considered editorially important are likely to be given greater prominence—placed on the front page, the front page of a section, or accompanied by a photograph [39]. In our study, among newspaper items, 106 (9.1%) appeared on the front page, 107 (9.2%) appeared on the first page of a section, and 170 (14.7%) had a photo accompanying the article (Table 1). Although a variety of employers were featured prominently, Weyco was most likely to receive prominent coverage, with 17 (12.0%) front page placements, 16 (18.3%) front page of section placements, and 26 (18.3%) accompanying photos.

### Reasons to adopt a hiring policy

Most news items (974, 84.0%) included one or more reasons why employers might decide to or had decided to hire only nonsmokers. Most could be considered business-related: reducing healthcare costs, increasing productivity, or following industry trends (Table 2). A remaining policy motivation commonly offered, healthier employees, was both health and business-related, as a healthier workforce was likely to incur fewer healthcare costs (Table 2). (Items did not typically specify whether a healthier workforce would be achieved by encouraging potential employees to quit smoking or by barring smokers from the pool of prospective employees.) Reducing healthcare costs was the most commonly cited reason to implement the policy (798, 68.9%), followed by healthier employees (600, 51.8%) (Table 2).

**Table 2:** Content of news items (n = 1,159) concerning employers with policies to hire only nonsmokers: United States, 1995–2013.

Content	Total	(%)	Example
*Reasons for adopting policy*			
Reduce healthcare costs	798	68.9	“It’s just a simple change in our hiring policy that would add a significant cost savings to taxpayers in Doylestown Township” [41].
Healthier employees	600	51.8	“We’re trying to have a healthier workforce” [42].
Absenteeism/productivity	309	26.7	“First, healthy employees are more productive employees” [43].
Industry trends	266	23.0	“The concept is inspired by a Michigan health-care company’s decision to prohibit smoking among all its employees” [44].
Other (positive role model/image, eliminate tobacco smoke)	13	1.1	“We want to be a good role model to our patients and the communities we serve” [45]. “Smoking is not the image that we want” [46].
*Arguments against policy*			
Discriminatory/violation of rights	709	61.2	“I just think it's discrimination … Same thing with having race issues, or if somebody's gay or obese or drinks coffee. What's the difference?” [47].
Slippery slope	418	36.1	“What personal habits or traits will this organization try to regulate next? Perhaps, obesity or alcohol use or the type of vehicle one drives or rides or the sports or hobbies one indulges in during personal time?” [48].
Tobacco a legal product	292	25.2	“What we’re talking about is whether employers should be making employment decisions based on the legal off-duty activities of their employees” [49].
Lose good employees	173	14.9	“If the University stopped hiring smokers, it might pass over potentially talented employees.” [50].
Unethical	4	0.3	“It is callous—and contradictory—for health-care institutions devoted to caring for patients regardless of the causes of their illness to refuse to employ smokers” [51].
*Employee responses to policy*	262	22.6	
Negative employee reaction	142	54.2	“I don’t believe any employer should be able to come in and tell you what you can do in your home” [52].
Positive employee reaction	68	26.0	“When the decision was announced to the medical staff, ‘they actually broke out in applause’” [53].
Neutral employee reaction	52	19.8	“It is normalized and not talked about much anymore because it’s part of the process” [54].
*Overall slant of opinion pieces*	406	35.0	
Positive	123	30.3	“The human and economic costs of tobacco use underscore the value of employers taking steps to encourage healthier habits among their employees” [55].
Negative	250	61.6	“How sad it is we have overzealous employers that are allowed to make policy that circumvents an individual’s rights and infringes on matters of personal yet legal activity away from the workplace” [56].
Mixed	33	8.1	“Whether employers should have the right to control private behavior of a legal product is questionable. The issue deserves a stiff civic debate. But what's interesting to me is that such restrictions are socially acceptable enough even to propose” [57].
*Overall slant of news stories*	753	65.0	
Positive	224	29.7	“The main thing is to keep a healthier employee. We get ‘em for 25 to 30 years or longer, and we want them to be healthy throughout their life while they’re here, as well as when they retire” [58].
Negative	93	12.4	“I think they should continue to do what they are so good at; giving care to the sick and leave the reforming of people’s habits to them” [59].
Neutral or mixed	436	57.9	“Is it really appropriate for a for-profit corporation to control what legal activities their employees engage in on their own time? … I'd have to say no. But it is appropriate to protect people in the company who don't want to be subjected to high or low levels of a toxic substance” [60].
*Experts cited/quoted*			
Civil liberties group mentioned	250	21.6	American Civil Liberties Union, National Workrights Institute [61]
Civil liberties group quoted	199	17.2	“There are a thousand things about people's private lives that employers don't like for a thousand different reasons” (Lewis Maltby, president of the National Workrights Institute) [61].
Tobacco control advocate/ organization/expert mentioned	187	16.1	Action on Smoking and Health [60]
Tobacco control advocate/ organization/expert quoted	96	8.3	“The overriding concern among employers is the recognition of just how expensive it is to have smokers as employees” (John Banzhaf, executive director of Action on Smoking and Health) [60].
Tobacco industry mentioned	91	7.9	Philip Morris, The Tobacco Institute [62]
Tobacco industry quoted	25	2.2	“But if people begin to assume that use of a product is injurious to their health—alcohol, salt, sugar and red meat—and then assume they have an obligation to sue the company that makes it, what we all will be left with is never-ending (litigation)” (Walker Merryman, vice president of The Tobacco Institute) [63].
*Evidence cited*			
Tobacco-related harm	577	49.8	“It is well-established that smoking and other uses of tobacco cause cancer, heart disease and lung disease” [64].
Economic toll of tobacco	322	27.8	“The U.S. Centers for Disease Control and Prevention estimates smoking costs $3,400 annually in excess medical costs and lost productivity per smoker” [65].
Laws governing hiring	617	53.2	“The resulting uproar led the Indiana Legislature to pass a law that forbids employers from discriminating against smokers” [63].

### Responses to the hiring policy

The majority of news items (829, 71.5%) mentioned one or more reasons to oppose a nonsmoker-only hiring policy. The most common objection was that the policy was discriminatory, punitive, or a violation of personal or civil rights (709, 61.2%) (Table 2). For example, in a 2005 *Vancouver* [Washington] *Columbian* newspaper article, a dismissed Weyco employee stated, “I don’t believe any employer should be able to come in and tell you what you can do in your home” [40]. Another common objection, mentioned in over one-third of items (418, 36.1%) (Table 2), was the “slippery slope” in hiring decisions that would inevitably follow, with employers refusing to hire candidates who were, for example, overweight or drank alcohol. News items rarely suggested that the policy was objectionable because employers would lose qualified employees (173, 14.9%), or because it was unethical (4, 0.3%) (Table 2).

Overall, most news stories conveyed a neutral or mixed impression of the policy (436, 57.9%); when a distinctively positive or negative slant was detected, it was more likely to be positive (224, 29.7%) than negative (93, 12.4%) (Table 2). However, employee reaction, when mentioned, was more likely to be negative (Table 2). Similarly, the majority of opinion pieces (letters-to-the-editor and editorials/op-eds) (250, 61.6%) expressed negative opinions of the policy, objecting on privacy or “slippery slope” grounds (Table 2). For example, a 2010 editorial in the *Doylestown* [Pennsylvania] *Intelligencer* noted that “if we’re worried about the rising healthcare costs in treating nicotine-related afflictions, we should be just as diligent in denying people employment because they eat Twinkies” [66]. However, among the handful of letters and columns written by tobacco control advocates, all but one supported the policy because it would promote employees’ smoking cessation or “healthy habits” [67–69], limit economic losses due to smokers’ higher medical costs and/or lower productivity levels [67–73], or denormalize smoking among youth [67]. The lone dissent came from the then-head of the American Legacy Foundation, Cheryl Healton, who, in a 2010 *El Paso* [Texas] *Times* guest column, described smoker-free hiring policies as “unjust” because smoking was concentrated among the economically disadvantaged who had the fewest resources to quit smoking [74].

### Expert opinion

In addition to citing public opinion, news items occasionally referred to or directly quoted various “experts.” The ACLU or other libertarian organizations were most commonly mentioned (250, 21.6%) and quoted (199, 17.2%) (Table 2); they invariably opposed the policy. Lewis Maltby of the National Workrights Institute (a spinoff of the ACLU) was frequently called on to represent the view that the policy was discriminatory, noting that “the number of things that we all do privately that have negative impact on our health is endless. If it’s not smoking, it’s beer” [75]. By contrast, the tobacco industry, which initiated and funded ACLU efforts to pass “smoker protection laws” [18, 19, 76], was mentioned infrequently (91, 7.9%) and quoted rarely (25, 2.2%).

Tobacco control advocates, organizations or experts were only occasionally mentioned (187, 16.1%) or directly quoted (96, 8.3%) (Table 2). Those who expressed an opinion were more likely to support (65/96, 67.7%) than oppose (29/96, 30.2%) or take no position on (2/96, 2.1%) the nonsmoker-only hiring policy. Action on Smoking and Health (typically represented by founder John Banzhaf) was frequently cited as a supporter of the policy (15 mentions), and offered the most consistent and clear rationale for its support: smokers were expensive employees, burdening employers with “up to $12,000 a year in additional costs” [77]. In the opposing camp, Michael Siegel, a professor at Boston University’s School of Public Health (23 mentions), described smoker hiring bans as “discriminatory” [78], “invasion[s] of privacy” [79], “punish[ing] smokers rather than helping them quit” [80], and opening the door to other forms of employment discrimination, such as refusing to hire the overweight [81, 82].

State and national American Cancer Society (ACS) and American Lung Association (ALA) offices were also frequently sought out for their perspectives (45 mentions); however, despite both organizations having adopted smoker hiring bans in the 1980s [83], they did not consistently support them. For example, in a 2005 article in the Lexington [Kentucky] *Herald-Leader*, an ALA Kentucky executive expressed opposition to firing smokers “because it has more to do with the bottom line, such as higher health insurance costs.” His organization preferred to “support an employee who is trying to quit smoking” [84]. A spokeswoman for the national office stated in 2008 that the ALA had no position on hiring bans; instead, its “main interest is in helping people quit and encouraging comprehensive smoke-free laws” [85]. The following year, the CEO of Florida’s ALA endorsed hiring bans, describing them as a “‘positive’ step that encourages people to give up smoking” [64]. By contrast, in 2012, an ALA spokesperson pointed out the absence of data “that proves nicotine-free hiring will encourage people to quit” and stated that “cessation programs are a more effective solution” [86]. The director of advocacy for ALA Ohio suggested that her organization might support smoker hiring bans in some cases: “[W]e don’t go out and say every company shouldn’t hire smokers; we promote the need for companies to give smokers the tools they need to stop” [87].

The ACS seemed similarly confused. In 1998, its cancer control director described a smoker hiring ban as “wonderful” because “if your job depends on it, maybe that will help convince people (not to smoke)” [88]. In 2002, commenting on St. Cloud, Florida’s decision to hire only nonsmokers, an ACS spokesperson described such policies as “harsh,” and said that ACS “prefers to promote cessation programs rather than punitive measures for smokers” [89]. Several days later, the Associated Press issued a correction; according to ACS Orlando’s executive director, the organization did, in fact, support St. Cloud’s policy [90]. Representatives of two local ACS chapters wrote to area newspapers to clarify that “ACS … supports St. Cloud in its decision to hire only nonsmokers” due to tobacco’s “financial costs” and because “smoking is the most preventable cause of death” [68, 69, 72].

### Evidence

Nearly half (577, 49.8%) of media items mentioned tobacco-related harm, disease (such as lung cancer and heart disease) or addiction (Table 2). Slightly more than one-quarter of items (322, 27.8%) mentioned tobacco’s economic toll, citing both national statistics on economic costs and employer-specific amounts (Table 2). For example, a 2012 news item in the *Columbia* [South Carolina] *Examiner* cited CDC statistics that smoking or exposure to secondhand smoke “costs the nation $193 billion in health bills and lost productivity” [91], while a 2005 *Associated Press* item noted that Union Pacific believed it would save “$922 annually for each position it fills with a nonsmoker over one who smokes” [92]. The majority of news items (617, 53.2%) also mentioned laws governing hiring and smokers, pointing out, for example, the legality of nonsmoker-only hiring policies, or mentioning the number of states that had “smoker protection” laws (Table 2).

## Discussion

Employers’ decisions to stop hiring smokers were ongoing newsworthy events, receiving sustained and sometimes prominent coverage over the 19-year period we examined, primarily in local newspapers in regions where such policies were enacted. Employers such as Weyco who fired employees who refused to be tested for nicotine received particular attention, perhaps because these stories had more newsworthy elements, most notably, individual human-interest drama; however, as the case of Geisinger Health Systems showed, even employers whose new policies did not result in firings might receive extensive coverage, suggesting that the topic sparked media interest.

News items justified nonsmoker-only hiring policies on both economic and health-related grounds; however, the most common justification was to reduce employers’ healthcare costs. This was a somewhat surprising finding. One might expect employers to emphasize concern for employees’ health over concerns for the bottom line, given the controversial nature of nonsmoker-only hiring policies and Americans’ broad disapproval of such policies. Concern for health would seem to be a more palatable public justification than cost savings for a policy that denies employment on the basis of behavior.

When news items did link the hiring policy to health promotion, they offered no evidence that these policies promoted health by, for example, encouraging smoking cessation. Instead, news items frequently cited evidence about tobacco-related harm, perhaps to provide some context for such policies, but without directly addressing their impact. News items also typically did not refer to expert opinion on the issue, opinion that might have shed more light on whether the policy was effective (or not) as a tobacco control measure. When they did, tobacco control advocates were rarely singled out as experts. Instead, journalists relied most often on civil liberties organizations such as the ACLU, which argued that the policies were discriminatory.

When the expert opinions of tobacco control advocates or organizations were sought, only two expressed clear, consistent arguments that drew on broader cultural values that might resonate with readers (i.e., equity, responsibility, fairness, and equality), the hallmark of successful advocacy [9, 12]. The two organizations most often sought out—ACS and ALA—had reached no consensus about whether to oppose, support, or, in the case of the ALA, take no position on nonsmoker-only hiring policies, despite having adopted such policies themselves. There was also no consensus about why these organizations took the stances they did, with different branches offering the same economic or health-based arguments both to support and oppose nonsmoker-only hiring policies.

Public health or tobacco control advocates were also largely absent from media coverage as authors, rarely writing letters or op-eds. Nearly all of those whose opinions were published supported the hiring policy; however, they tended to emphasize economic over health arguments as the reason for their support. Only one tobacco control advocate voiced one of the ethical objections to nonsmoker-only hiring policies raised within public health circles, pointing out that such policies disproportionately affected an already disadvantaged population. Overall, the media rarely referenced any ethical concerns about the policy.

## Conclusion

As presented in the media, nonsmoker-only hiring policies appeared to have little to do with tobacco control, as economic concerns were a primary driver and no evidence was presented to establish that these policies reduced smoking prevalence or were more effective in doing so than other policies. Perhaps due to the focus on business cost savings, tobacco control advocates were not considered natural experts on the issue; despite or perhaps because of internal debates about nonsmoker-only hiring policies, they took public positions on this issue very infrequently.

Although our results indicate that the media’s interest in the issue potentially offers opportunities for tobacco control advocates to engage the media, the status quo, in which the economics of nonsmoker only hiring policies is primarily emphasized, has the advantage of distancing tobacco control from a policy highly unpopular with the public [22]. Rather than being regarded as another “nanny state” intervention, such policies are seen as business decisions. Given that the evidence is also unclear as to whether such policies actually are effective from a health standpoint, staking a claim to this issue may do little to advance the larger tobacco control agenda.
